# P-1755. Developing Host-based Signatures of Gram-negative Infection to Guide Empiric Antibiotics for Sepsis

**DOI:** 10.1093/ofid/ofaf695.1926

**Published:** 2026-01-11

**Authors:** Alyse Wheelock, Elizabeth Chiyka, Te Vantha, George Oduro, Yasith Ros, Vannsay Yin, Chris Oppong, Yaw Agyekum Boaitey, Alex Owusu-Ofori, Nehkonti Adams, Deborah Striegel, Patrick Blair, Joshua Chenoweth, Danielle Clark

**Affiliations:** Austere environments Consortium for Enhanced Sepsis Outcomes, The Henry M. Jackson Foundation for the Advancement of Military Medicine, Bethesda, Maryland; Austere environments Consortium for Enhanced Sepsis Outcomes, The Henry M. Jackson Foundation for the Advancement of Military Medicine, Bethesda, Maryland; Takéo Provincial Hospital, Takéo Province, Takeo, Cambodia; Komfo Anokye Teaching Hospital, Accra, Greater Accra, Ghana; Lucerent Clinical Solutions Limited, Takeo, Takeo, Cambodia; Lucerent Clinical Solutions Limited, Takeo, Takeo, Cambodia; Komfo Anokye Teaching Hospital (KATH), Kumasi, Ashanti, Ghana; Komfo Anokye Teaching Hospital (KATH), Kumasi, Ashanti, Ghana; Komfo Anokye Teaching Hospital,, kumasi, Central, Ghana; Naval Medical Research Center Infectious Diseases Directorate, Bethesda, Maryland; Henry M. Jackson Foundation, Bethesda, Maryland; Austere environments Consortium for Enhanced Sepsis Outcomes, The Henry M. Jackson Foundation for the Advancement of Military Medicine, Bethesda, Maryland; Austere environments Consortium for Enhanced Sepsis Outcomes, The Henry M. Jackson Foundation for the Advancement of Military Medicine, Bethesda, Maryland; The Henry M. Jackson Foundation for the Advancement of Military Medicine, Inc., Bethesda, MD, Bethesda, Maryland

## Abstract

**Background:**

Mounting evidence indicates that broad-spectrum antibiotic overuse is widespread in the management of suspected sepsis and leads to harms. Clinicians lack timely diagnostic tools to guide when broad empiric gram-negative (GN) coverage is needed. To address this gap, we aimed to identify a host-based signature of GN infection as a proof-of-concept for development of a future rapid diagnostic assay.

**Table 1.**
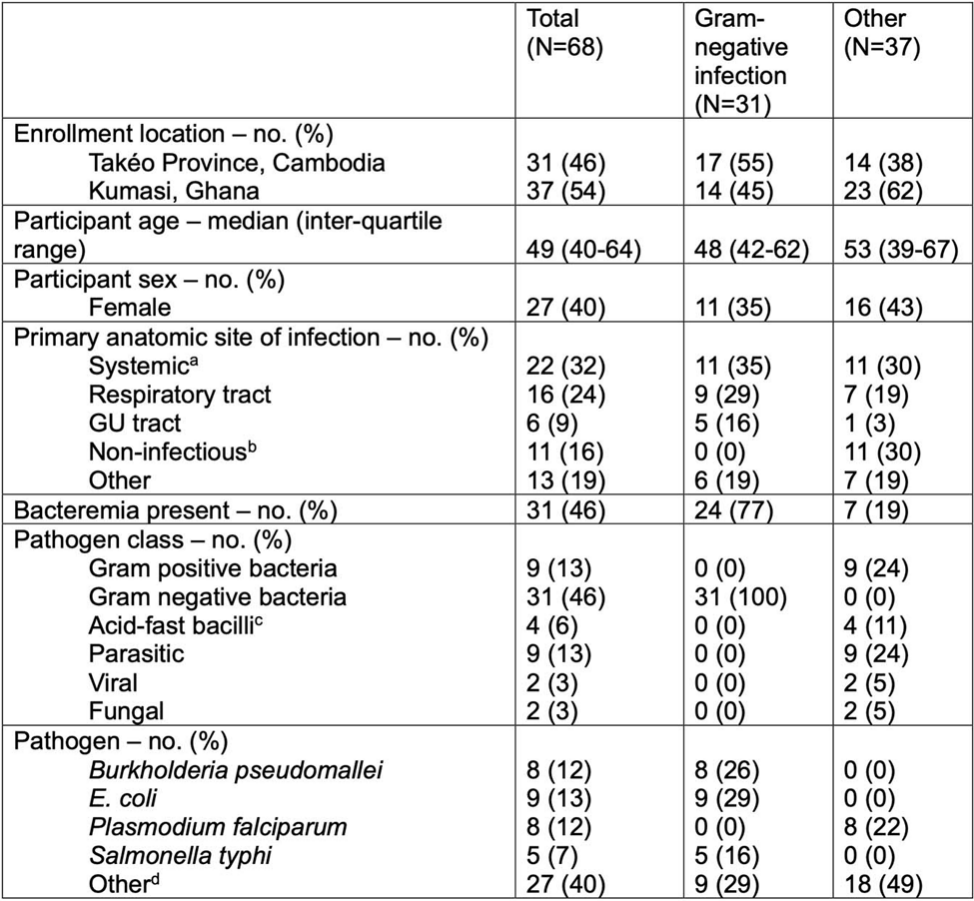
Participant and pathogen characteristics in classified discovery/cross-validation dataset a. “Systemic infections” refers to processes without a primary anatomic site of infection, such as dengue, but not to cases where bacteremia complicated a primary site of infection. b. Participants who presented with suspected sepsis and met enrollment criteria but were ultimately adjudicated as having a confirmed non-infectious disease, e.g. congestive heart failure or diabetic ketoacidosis. c. All were cases of disease caused by Mycobacterium tuberculosis. d. Other pathogens included Streptococcal spp. (n=5), Staphylococcus aureus (n=4), Mycobacterium tuberculosis (n=4), dengue virus (n=2), among other pathogens.

**Figure:**
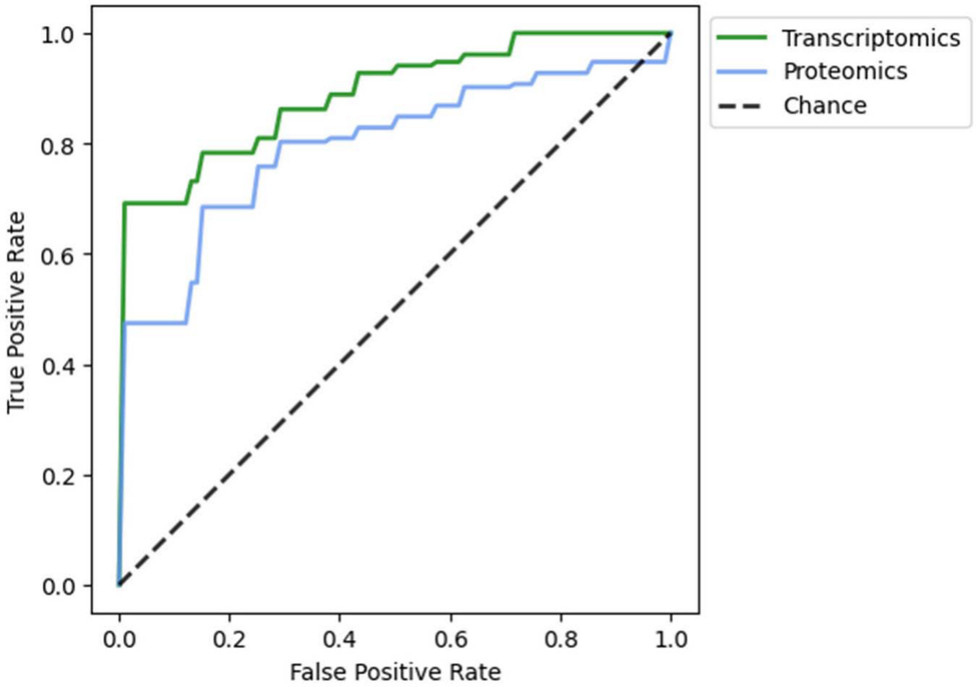
Performance of host transcript- and protein-based signatures of gram negative infection in cross-validation Area under the receiver operating characteristic curves for gram-negative signatures compared to adjudicated diagnoses, based on stratified 5-fold cross-validation with 5 repeats.

**Methods:**

This analysis utilized RNASeq and a 29-analyte proteomics dataset derived from blood collected from participants at time of enrollment into a longitudinal study of sepsis conducted by the Austere environments Consortium for Enhanced Sepsis Outcomes (ACESO). Enrollment criteria in the original study were based on sepsis-2 definitions. Participants from sites in Kumasi, Ghana and Takéo Province, Cambodia who had confirmed, independently adjudicated diagnoses were included in this analysis. The dataset was classified into participants with laboratory confirmed GN infection and participants with any other non-GN confirmed diagnosis. We performed t-tests with False Discovery Rate correction, followed by an exhaustive search of transcript and protein combinations using logistic regression with a stratified 5-fold cross-validation with 5 repeats.

**Results:**

The discovery/cross-validation dataset included 31 GN infections and 37 other cases. A transcriptomics exhaustive search yielded a signature featuring SMARCD3, EIF4G1, and IER5 with an area under the receiver operating characteristic (AUROC) curve of 0.891. A protein-based signature (features: IL17A, IL1RA, and MMP8) had an AUROC of 0.795.

**Conclusion:**

The approach used in this exploratory analysis shows promise for development of a rapid test to guide empiric antibiotics for sepsis while culture-based diagnostics are pending. This rapid test would provide critical benefit to patients in limited-resource settings, including in low- and middle-income countries, pandemic and mass-casualty scenarios, and prolonged field care during military operations. Further work is needed, first to expand the discovery dataset to ensure selection of features that will perform robustly across epidemiologic settings, and second to formally validate the signature in a separate test set.

**Disclosures:**

All Authors: No reported disclosures

